# Autocrine Activation of the Wnt/β-Catenin Pathway by CUX1 and GLIS1 in Breast Cancers

**DOI:** 10.1242/bio.20148193

**Published:** 2014-09-12

**Authors:** Charles Vadnais, Peiman Shooshtarizadeh, Charles V. Rajadurai, Robert Lesurf, Laura Hulea, Sayeh Davoudi, Chantal Cadieux, Michael Hallett, Morag Park, Alain Nepveu

**Affiliations:** 1 Goodman Cancer Research Centre, McGill University, Montreal, QC H3A 1A3, Canada; 2 Department of Biochemistry, McGill University, Montreal, QC H3G 1Y6, Canada; 3 Department of Medicine, McGill University, Montreal, QC H3A 1A1, Canada; 4 Department of Oncology, McGill University, Montreal, QC H2W 1S6, Canada; 5 McGill Centre for Bioinformatics, McGill University, Montreal, QC H3G 0B1, Canada

**Keywords:** Breast cancer, Expression profiling, Mouse tumor model, Transcriptional regulation, Wnt/β-Catenin pathway

## Abstract

Autocrine activation of the Wnt/β-catenin pathway occurs in several cancers, notably in breast tumors, and is associated with higher expression of various Wnt ligands. Using various inhibitors of the FZD/LRP receptor complex, we demonstrate that some adenosquamous carcinomas that develop in MMTV-CUX1 transgenic mice represent a model for autocrine activation of the Wnt/β-catenin pathway. By comparing expression profiles of laser-capture microdissected mammary tumors, we identify Glis1 as a transcription factor that is highly expressed in the subset of tumors with elevated *Wnt* gene expression. Analysis of human cancer datasets confirms that elevated *WNT* gene expression is associated with high levels of CUX1 and GLIS1 and correlates with genes of the epithelial-to-mesenchymal transition (EMT) signature: *VIM*, *SNAI1* and *TWIST1* are elevated whereas *CDH1* and *OCLN* are decreased. Co-expression experiments demonstrate that CUX1 and GLIS1 cooperate to stimulate TCF/β-catenin transcriptional activity and to enhance cell migration and invasion. Altogether, these results provide additional evidence for the role of GLIS1 in reprogramming gene expression and suggest a hierarchical model for transcriptional regulation of the Wnt/β-catenin pathway and the epithelial-to-mesenchymal transition.

## INTRODUCTION

The Wnt/β-catenin signaling pathway plays an important role in the development and regeneration of several tissues by stimulating the growth of stem cells and multipotential progenitors (MacDonald et al., 2009; Nusse, 2008). Wnts are secreted extracellular proteins that trigger a wide range of cellular responses upon receptor binding and activation. Most organisms contain multiple Wnt genes which initiate distinct cellular pathways, namely the canonical Wnt/β-catenin pathway, the planar cell polarity pathway, or the Wnt/calcium pathway. The β-catenin protein is maintained in a cytoplasmic complex that includes APC, Axin, and GSK3β. Phosphorylation by GSK3β causes the degradation of β-catenin unless the Wnt/β-catenin pathway is activated by Wnt ligands. Wnt proteins initiate the canonical pathway by binding and activating the Frizzled (Fzd) and LRP5/6 receptors. Activation of these receptors induces the dissociation of β-catenin from the degradation complex. As its concentration increases, β-catenin moves to nucleus where it displaces the Groucho corepressor from the TCF/Lef family of transcription factors with which it functions as a co-activator. As a consequence, the TCF/β-catenin complex now activates genes that were previously repressed by the TCF/Groucho complex. Many of the TCF/β-catenin target genes code for Wnt signaling components that are capable of enhancing or antagonizing Wnt pathway activity (MacDonald et al., 2009). Therefore, its own targets can regulate the Wnt pathway through feedback loop mechanisms (He et al., 1998). Activation of the FZD and LRP5/6 receptors can be inhibited by two types of secreted proteins. Secreted Frizzled-related proteins (sFRPs) and the Wnt inhibitory factor (WIF) bind Wnt proteins and prevent their interaction with LRP5/6 receptors, whereas Dickkopf (DKK) and sclerostin (SOST) compete with Wnt for binding to LRP5 and LRP6 (Dann et al., 2001; Hoang et al., 1996; Hsieh et al., 1999; Semenov and He, 2006).

Much of our knowledge of the Wnt pathway was originally derived from studies in *drosophila*. Failure to express either *wingless* or *cut* in the wing margin causes similar phenotypes from which their names were derived: *wingless* and *cut* wing (Couso et al., 1994; de Celis and Bray, 1997; Micchelli et al., 1997; Neumann and Cohen, 1996). In the wing margin, *cut* is required in a cell-autonomous manner for wingless expression, whereas *wingless* is required for *cut* expression in neighboring cells (Micchelli et al., 1997). Orthologs of *cut* in mammals are Cut homeobox 1 and 2, CUX1 and CUX2. In *Drosophila* imaginal discs, wingless is directly regulated by Cubitus interruptus, Ci, a transcription factor whose regulatory activity is under the control of the hedgehog ligand, Hh (Chen et al., 2000; Von Ohlen and Hooper, 1997). In mammals, there are several orthologs of Ci: Gli1, Gli2, Gli3, Glis1, Glis2 and Glis3 (Lichti-Kaiser et al., 2012; Riobo and Manning, 2007). Both Gli1 and Gli2 have been implicated in human cancers (Dahmane et al., 1997; Sheng et al., 2002). There is evidence to show that the Wnt/β-catenin pathway can be stimulated by Gli transcription factors, whether through activation of Wnt gene expression (Mullor et al., 2001), or indirectly via induction of Snail and downregulation of E-cadherin (Li et al., 2007). CUX1 was also shown to activate Snail gene expression and to cooperate with Snail in the repression of the E-cadherin gene (Kedinger et al., 2009).

*CUX1* encodes several isoforms that exhibit strikingly different DNA binding properties (reviewed in (Nepveu, 2001; Sansregret and Nepveu, 2008)). The full-length protein, often called p200 CUX1, is very abundant, binds rapidly but only transiently to DNA and functions as an accessory factor in base excision repair (Ramdzan et al., 2014). The shorter isoforms p75 and p110 CUX1 bind stably to DNA and function as transcriptional repressors or activators depending on the promoter (Goulet et al., 2002; Harada et al., 1995; Harada et al., 2008; Moon et al., 2000; Moon et al., 2001). The p75 and p110 CUX1 isoforms exhibit similar DNA binding transcriptional activities (Cadieux et al., 2009; Goulet et al., 2002; Kedinger et al., 2009).

Cell-based assays demonstrated a role for *CUX1* in cell cycle progression and cell proliferation (Sansregret et al., 2006; Truscott et al., 2007), strengthening of the spindle assembly checkpoint (Sansregret et al., 2011), cell migration and invasion (Kedinger et al., 2009; Michl et al., 2005), the DNA damage response (Vadnais et al., 2012), resistance to apoptotic signals (Ripka et al., 2010), as well as dendrite branching and spine development in cortical neurons (Cubelos et al., 2010) (reviewed by Hulea and Nepveu, 2012).

In colon cancer, the Wnt/β-catenin pathway is frequently activated following inactivation of the tumor suppressor APC or mutations in the β-catenin or Axin genes (Segditsas and Tomlinson, 2006). Recently, another mechanism of Wnt/β-catenin pathway activation was demonstrated where transcriptional activation of one or several of the Wnt genes leads to autocrine activation of Frizzled and LRP receptors and subsequent increase in nuclear β-catenin (Bafico et al., 2004). This autocrine activation has been shown in a sizeable proportion (20–25%) of breast cancers (Bafico et al., 2004; Benhaj et al., 2006; Schlange et al., 2007), lung cancers (Akiri et al., 2009), neuroblastomas (Liu et al., 2008), acute myeloid leukemias and myelodysplastic syndromes (Xu et al., 2008). Only a few transcription factors so far have been implicated in transcriptional regulation of the Wnt/β-catenin pathway, including factors of the Gli family, CUX1 and ATF3 (Cadieux et al., 2009; Chen et al., 2000; Fiaschi et al., 2009; Grove et al., 1998; Micchelli et al., 1997; Von Ohlen and Hooper, 1997; Yan et al., 2011).

Transgenic mice expressing the p75 or p110 CUX1 isoform under the control of the mouse mammary tumor virus (MMTV) regulatory sequences were previously shown to develop mammary tumors of diverse histologic types (Cadieux et al., 2009). Interestingly, approximately one third of the mammary tumors resemble tumors previously characterized in MMTV-Wnt1 transgenic mice and were classified as adenosquamous carcinomas (Li et al., 2000). Analysis of 6 of these adenosquamous carcinomas revealed the presence of β-catenin in the nucleus and increased expression of either WNT1, WNT6, WNT8B and WNT10A or combination thereof. Evidence from multiple experimental approaches indicated that CUX1 is directly involved in the regulation of several Wnt genes. RNAi-mediated CUX1 knockdown caused a decrease in the expression of WNT1, WNT6, WNT8B and WNT10A, while chromatin immunoprecipitation and reporter assays demonstrated that p75 and p110 CUX1 bind to and activate the promoter of each of these genes (Cadieux et al., 2009). These findings led us to formulate two hypotheses. First, that MMTV-CUX1 mammary tumors with high WNT expression represent a model for autocrine activation of the Wnt/β-catenin pathway. Secondly, since only one third of the mammary tumors in MMTV-CUX1 transgenic mice exhibit high WNT expression, we hypothesized that other factors are needed, in addition to CUX1, for transcriptional activation of this pathway. In the present study, we employed various types of inhibitors to establish that activation of the FZD/LRP receptor complex is required for the augmentation of TCF/β-catenin transcriptional activity observed in MMTV-CUX1 mammary tumor cells. Furthermore, by comparing the expression profiles of laser-capture microdissected tumor cells from a panel of randomly selected MMTV-CUX1 mammary tumors, we identified GLIS1 as a transcription factor that is significantly overexpressed in tumors with high WNT gene expression. Co-expression experiments in MCF10A untransformed mammary epithelial cells demonstrated that CUX1 and GLIS1 cooperate in the autocrine activation of the Wnt/β-catenin pathway and in stimulating cell migration and invasion.

## MATERIALS AND METHODS

### Cell Culture

All lines were maintained in Dulbecco's modified minimum essential medium (DMEM) supplemented with penicillin/streptomycin, glutamine, and 10% fetal bovine serum (all from Invitrogen). Cells were cultured in a humidified incubator at 37°C and 5% CO_2_.

### Protein Extracts

Nuclear extracts were prepared using a procedure adapted from Lee et al. (Lee et al., 1988). Briefly, cells were submitted to three freeze/thaw cycles in Buffer A (10 mM Hepes, pH 7.9, 10 mM KCl, 1.5 mM MgCl_2_, 1 mM DTT). Nuclei were then resuspended in Buffer C (20 mM Hepes, pH 7.9, 25% glycerol 1.5 mM MgCl_2_, 420 mM NaCl_2_, 0.2 mM EDTA) and incubated at 4°C for 30 min. After 15 min of centrifugation, the supernatant was collected. Buffers A and C were supplemented with protease inhibitor mix tablet (Roche Applied Science).

### Immunoblotting

Proteins extracts were resuspended in Laemmli buffer, boiled for 5 min, resolved by SDS-PAGE, and electrophoretically transferred to a PVDF membrane. Membranes were blocked in TBS-T (10 mM Tris, pH 8, 150 mM NaCl, 0.1% Tween X-100) containing 5% milk and 2% bovine serum albumin. Membranes were then incubated with primary antibodies diluted in TBS-T, washed in TBS-T, and incubated with species-specific secondary antibodies conjugated to horseradish peroxidase for 45 min at room temperature. Proteins were then visualized using the ECL system of Amersham Biosciences according to the manufacturer's instructions. The following antibodies and dilutions were used: Cux1 (Moon et al., 2001) (#1142), γ-tubulin (1:15,000, Sigma), α-actin (1:10,000, Santa Cruz) β-catenin (1:2000, BD Biosciences).

### siRNA knockdown

CUX1 knockdown was performed by transfecting cells with a pair of siRNA constructs specific for CUX1 mRNA (5′ GAAUCUUCUCGUUUGAAACUUUGAA and 5′ GCUUCAGAGCGAUAAUACACUAUUA) using Lipofectamine2000 (Invitrogen) according to the manufacturer's instructions.

### Lentiviral constructions

The lentivirus vectors were constructed by inserting the p110 CUX1 sequences into a pRev vector containing Hygromycin resistance and the Glis1 sequence into a pLenti6 vector containing a Puromycin resistance gene. Lentiviruses were produced by transient transfection of 293FT cells by using Lipofectamine 2000 according to the manufacturer's protocol (Invitrogen).

### RNA extraction and Real Time PCR

RNA was extracted using TRIzol reagent (Invitrogen), and cDNA was prepared using Superscript II RNase H-reverse transcriptase kit (Invitrogen) following the manufacturer's instructions. Real time PCR was performed on a LightCycler instrument using the FastStart DNA Master SYBR Green kit (Roche Applied Science) and specific primer pairs for each gene.

### Luciferase assays

Luciferase assays were performed as previously described (Cadieux et al., 2009) with minor modifications in HS578T and HEK293T cells. For TOP/FOP reporter assay, Cells plated at 8×10^4^ per well in 12-well plates were cotransfected with 0.25 µg of either the TOP 8× or FOP 8× reporter plasmids (Addgene) and 0.5 µg of either CUX1 p110 or empty vector as effector. As a control for transfection efficiency, the β-galactosidase protein (Sigma) was included in the transfection mix and the luciferase activity was normalized based on β-galactosidase activity.

### Laser-Capture-Microdissection

Tumors from the previously generated p75-CUX1 and p110-CUX1 transgenic mice (Cadieux et al., 2009) were embedded in Tissue Freezing Medium (Triangle Biomedical Sciences #TFM-5) and flash frozen in liquid nitrogen. 10 µm slices of tissue were prepared in a Microm HM505E Cryostat at ∼−30°C on positively charged slides (Fisherbrand Superfrost/Plus #12-550-15). Slides were then stained using a shortened H&E protocol (Ponzo et al., 2009). Epithelial cells were then isolated by IR (InfraRed) pulse on an Arcturus XT Laser Capture Microdissection instrument within 30–45 minutes of staining to obtain 100–150 pulses of material of 20–25 µm in diameter. RNA was isolated from the microdissection caps using the Picopure RNA isolation kit from Arcturus according to the manufacturer's instructions, including the optional DNAse treatment using the Qiagen RNase-Free DNase kit (#79254).

### Expression Profiling

RNA isolated from microdissected tissue was amplified using the Arcturus RiboAmp HS PLUS RNA amplification kit according to the manufacturer's instructions for 2 rounds of amplification. Amplified mRNA (aRNA) was labeled using the Arcturus Turbo Labeling Cy5 and Cy3 kits using slightly modified instructions: The labeling reaction was carried out using 5 µg of aRNA in a 20 µl volume instead of 50 µl to increase the dye incorporation rate. Labeled aRNA was hybridized to Agilent's Whole Mouse Genome Microarray (G4112F), washed and scanned on an Agilent 5 µm scanner model G2505B according to the manufacturer's instructions.

### Expression profiling data analysis

Expression profiling array images were processed using the Feature Extraction software from Agilent. The raw data was then analyzed using the LIMMA (Smyth, 2005) package on the R platform (http://www.r-project.org). The “gplots” R package was also used.

### Public dataset recovery and Meta-analysis

Human breast cancer expression profiling datasets were obtained from the Oncomine database (https://www.oncomine.org). We retrieved all datasets comprising 100 or more samples and for which expression of at least one of CUX1 and GLIS1 as well as at least 15 out of the 19 Wnt ligand genes were available (As of November 2012). Note that datasets using Affymetrix platforms contain probes for the CASP transcript at the CUX1 locus. For each dataset, samples were sorted from lowest to highest expression of Wnt ligand genes using the BreSAT algorithm (see below). The 25% highest Wnt expressing samples were compared to the 25% lowest Wnt expressing samples for expression of CUX1, Glis1 and the EMT markers Cdh1, Cdh2, Ocln, Snai1, Snai2, Vim and Twist.

### Breast Signature Analysis Tool (BreSAT)

This algorithm is a semi-parametric method for linearly ordering samples according to their expression of a given set of genes. It operates similarly to the initial steps of the U-statistic (Abraham et al., 2010; Wilcoxon, 1945), by independently computing the ranks for each gene across all samples and then summing the ranks within each sample. Because samples are not assigned a priori to discrete classes, the algorithm then linearly reorders the samples according to the ascending sum of these ranks (Lesurf, 2008) (http://digitool.library.mcgill.ca/R/-?func = dbin-jump-full&object_id = 32499&silo_library = GEN01).

### Live-cell Imaging

1×10^4^ cells were plated on 10 µg/ml fibronectin coated plates for three hours and then subjected to time lapse-video microscopy with a frame taken every 5 minutes for a total of 16 hrs. The last forty frames from each condition were subjected to analysis using the Metamorph software.

### Invasion assays

Modified Boyden chambers with 8 mm pore size were coated with a mixture of Matrigel (BD biosciences) with final concentration of 1.25 mg/ml and acid purified Rat tail Collagen I (Gibco) with final concentration of 2.5 mg/ml for 2 hrs at 37°C. Cells (1×10^6^) were seeded on the bottom of the filters and were incubated at 37°C with 5% CO_2_ for 5 hrs to adhere. After 5 hrs, filters were inverted and top compartment was filled with 10% FBS and the bottom compartment were filled with plain DMEM and cells were allowed to invade for 48 hrs towards serum. Cells were stained with Calcein-AM (Invitrogen) as per the manufacturer's instructions and confocal Z-stacks were acquired every 10 µm. Cells invading 20 µm into the matrix and beyond were quantified as percentage of total cells.

## RESULTS

### Elevated Wnt Gene Expression in Mammary Tumor Cells Leads to Autocrine Activation of the Wnt/β-catenin Pathway

**Fig. 1.**
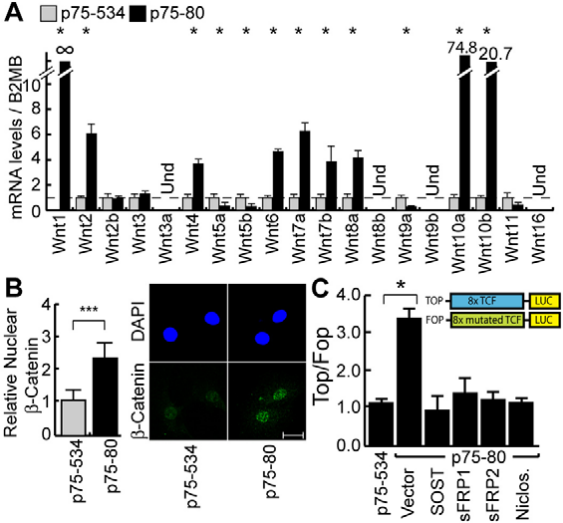
Autocrine Activation of the Wnt/β-Catenin Pathway in Some Mammary Tumors from MMTV-CUX1 Transgenic Mice. Cell lines were established from two mammary tumors that developed in transgenic mice carrying a MMTV-p75 CUX1 transgene: an adenosquamous carcinoma from mouse #p75-80 and a solid carcinoma from mouse #p75-534. The two cell lines were analyzed for Wnt gene expression (A), nuclear β-catenin expression (B), and TCF/β-catenin transcriptional activity (C). (A) mRNA levels of Wnt ligand genes were measured by RT-qPCR. Results of triplicate experiments are shown. * P Value<0.05 on a Welch corrected T test. Und: Undetected. (B) Cells were analyzed by indirect immunofluorescence using antibodies specific for the active, non-phosphorylated form of β-Catenin. Histogram shows the relative mean nuclear signal for β-Catenin as measured using the ImageJ software. The value for p75-534 was set to 1. * P Value<0.05 on a Welch corrected T test. A representative image of each cell line is shown on the right. Scale bar: 20µm. (C) The TOP/FOP luciferase reporter assay was performed in the p75-534 and p75-80 mammary tumor cell lines. In addition, p75-80 cells were cotransfected with a vector expressing either sFRP1, sFRP2 or SOST or an empty vector. Where indicated, niclosamide was added to the cells at the time of transfection. Results of 3 independent transfections are shown. * P Value<0.05 on a Welch corrected T test. A schematic representation of the reporter constructs is shown.

To be able to investigate whether elevated Wnt gene expression in MMTV-CUX1 mammary tumors leads to autocrine activation of the Wnt/β-catenin pathway, we first established cell lines from the p75-80 adenosquamous carcinoma and the p75-534 solid carcinoma. The numbers p75-80 and p75-534 refer to the particular tumor bearing mice in the transgenic mouse cohort expressing the p75 CUX1 isoform under the control of MMTV regulatory sequences, as detailed in the introduction. Based on the histological features of these tumors, we predicted that cell lines derived from these tumors would respectively express high and low levels of Wnt genes (Cadieux et al., 2009). We found out later that the correlation between histological types and Wnt gene expression is not absolute (see below, Fig. 4). Nevertheless, RT-qPCR analysis indicated that expression of Wnt1, Wnt2, Wnt4, Wnt6, Wnt7a, Wnt7b, Wnt8a and especially Wnt10a and Wnt10b was much higher in the p75-80 than in the p75-534 cell line (Fig. 1A). In addition, immunofluorescence using an antibody specific for the active form of the β-catenin protein showed higher nuclear levels in the p75-80 line (Fig. 1B). As a functional assay to monitor the transcriptional activity of TCF/β-catenin complexes in these two cell lines, we measured the expression ratio of the TOP and FOP luciferase reporter plasmids. The two plasmids are identical except for the presence of 8 TCF binding sites in the TOP reporter, whereas these sites are inactivated in the FOP reporter. The TOP reporter, was activated in the p75-80 but not in the p75-534 cell line, confirming that the β-catenin pathway is activated in the former (Fig. 1C). Importantly, activation of the TOP reporter in p75-80 cells was prevented in the presence of niclosamide, or upon cotransfection of plasmids expressing sFRP1, sFRP2 or SOST (Fig. 1C). As niclosamide promotes Frizzled1 internalization and down-regulates Dvl-2 expression (Chen et al., 2009b), whereas sFRP1/2 and SOST/DKK inhibit activation of the FRP-LRP5/6 receptor by respectively binding to Wnt ligands or the LRP5/6 receptor (Dann et al., 2001; Hoang et al., 1996; Hsieh et al., 1999; Semenov and He, 2006), these results indicate that elevated TCF/β-catenin transcriptional activity in p75-80 cells results from autocrine activation of the Wnt pathway.

### CUX1 Is Required for Maximal Expression of the Wnt Genes in Human Tumor Cell Lines

**Fig. 2.**
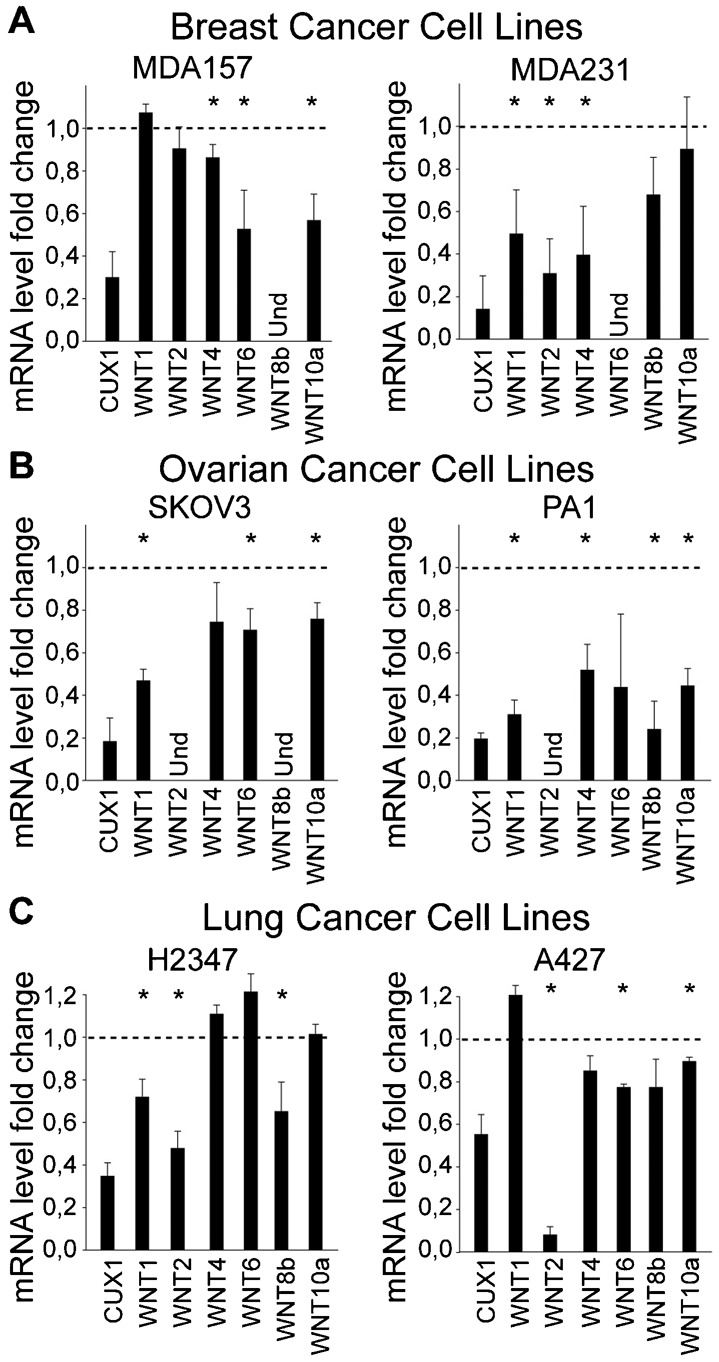
CUX1 Is Required for Maximal Expression of Wnt Genes in Human Tumor Cell Lines. CUX1 specific or scrambled siRNA were transfected in a panel of 6 human cancer cell lines from breast (A), ovarian (B) and lung (C) cancers. 3 days later total mRNA was isolated and quantitative RT-PCR was performed using HPRT1 mRNA as a control. The values represent fold difference in mRNA expression between cells treated with CUX1 or scrambled siRNA. (Und: undetected). * P Value<0.05 on a Welch corrected T test.

A number of human cancer cell lines have been reported to display autocrine activation of the Wnt/β-catenin pathway (Akiri et al., 2009; Bafico et al., 2004; Vijayakumar et al., 2011). To verify whether CUX1 is required for expression of Wnt genes in these human cancer cells, we performed siRNA-mediated knockdown of CUX1 in six cell lines, two each from breast (MDA157 and MDA231), ovarian (PA-1 and SKOV3) and lung (H2347 and A427), and we performed RT-PCR analysis to measure the expression of Wnt genes previously identified as transcriptional targets of CUX1 in chromatin immunoprecipitation (ChIP) assays: Wnt 1, 2, 4, 6, 8b and 10a (Cadieux et al., 2009; Harada et al., 2008; and unpublished observations). In every cell line, CUX1 knockdown caused a significant decrease in the expression of three or more *WNT* genes (Fig. 2). We conclude that CUX1 is required for maximal expression of *WNT* genes in human tumor cell lines that display autocrine activation of the Wnt/β-catenin pathway.

### Ectopic Expression of p110 CUX1 Leads to Autocrine Activation of the Wnt/β-Catenin Pathway in Human Tumor Cell Lines

**Fig. 3.**
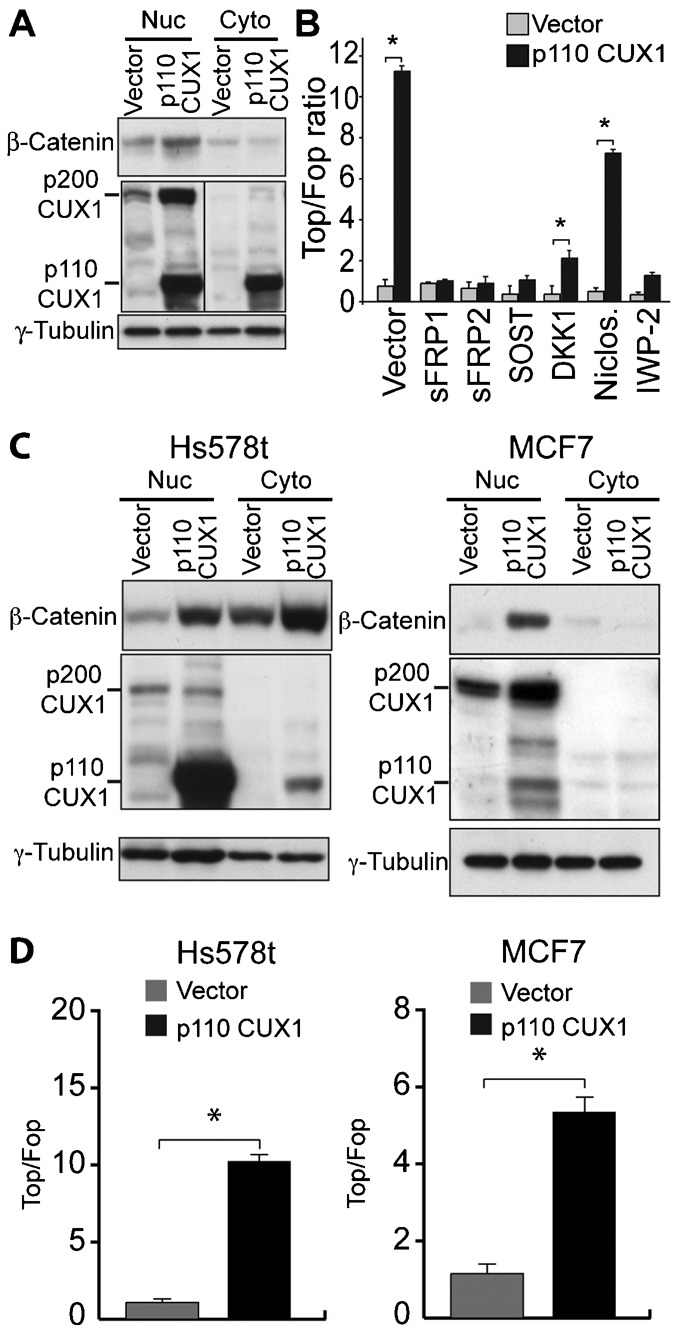
Ectopic Expression of p110 CUX1 Leads to Autocrine Activation of the Wnt/β-Catenin Pathway in Human Tumor Cell Lines. (A) HEK293 cells were infected with retroviruses to establish cells stably carrying a vector that expresses p110 CUX1 or nothing (vector). Nuclear and cytoplasmic extracts were analyzed by immunoblotting with the indicated antibodies. (B) The TOP or FOP luciferase reporters were introduced into HEK293 cells together with 500 ng of a vector expressing p110 CUX1 or an empty vector. Luciferase activity was measured 36 hours after transfection. Where indicated, a vector expressing either sFRP1, sFRP2, SOST or DKK1 was transfected as well, or niclosamide or IWP-2 was added to the cells at the time of transfection. Results of 3 independent transfections are shown. * P Value<0.05 on a Welch corrected T test. (C) Hs578t and MCF7 breast cancer cells were stably infected with either a lentivirus expressing p110 CUX1 or an empty lentivirus (vector). Nuclear and cytoplasmic extracts were analyzed by immunoblotting with the indicated antibodies. (D) The TOP or FOP luciferase reporters were introduced into Hs578t and MCF7 cells together with 500 ng of a vector expressing p110 CUX1 or an empty vector. Luciferase activity was measured 36 hours after transfection.* P Value<0.05 on a Welch corrected T test.

We next verified whether ectopic CUX1 expression would activate the Wnt/β-catenin pathway in cell lines that do not display autocrine activation of this pathway. Nuclear β-catenin expression was significantly higher in HEK293 cells stably expressing p110 CUX1 (Fig. 3A). In agreement with these results, co-expression of p110 CUX1 with the TOP and FOP reporter plasmids led to a striking elevation in the TOP/FOP ratio (Fig. 3B). Similar increases in nuclear β-catenin expression and TCF/β-catenin transcriptional activity were observed in Hs578T and MCF-7 human breast cancer cells upon ectopic expression of p110 CUX1 (Fig. 3C,D). Importantly, co-expression of sFRP1, sFRP2, SOST or DKK1 or transfection in the presence of the niclosamide or IWP-2 inhibitors in HEK293 cells in every case annihilated the stimulatory effect of p110 CUX1 (Fig. 3B). As mentioned earlier, sFRP1/2, SOST, DKK and niclosamide inhibit the Wnt/β-catenin pathway by preventing activation of the FRP-LRP5/6 receptor. In addition, IWP-2 blocks signaling by Wnt ligands by inhibiting Porcupine (Porcn), which is required for palmitoylation and subsequent secretion of Wnt ligands (Chen et al., 2009a). Together, these results demonstrate that p110 CUX1 can activate the Wnt/β-catenin pathway through an autocrine loop.

### Activation of the Wnt/β-Catenin Pathway in MMTV-CUX1 Mammary Tumors Is Associated with High Expression of Glis1

**Fig. 4.**
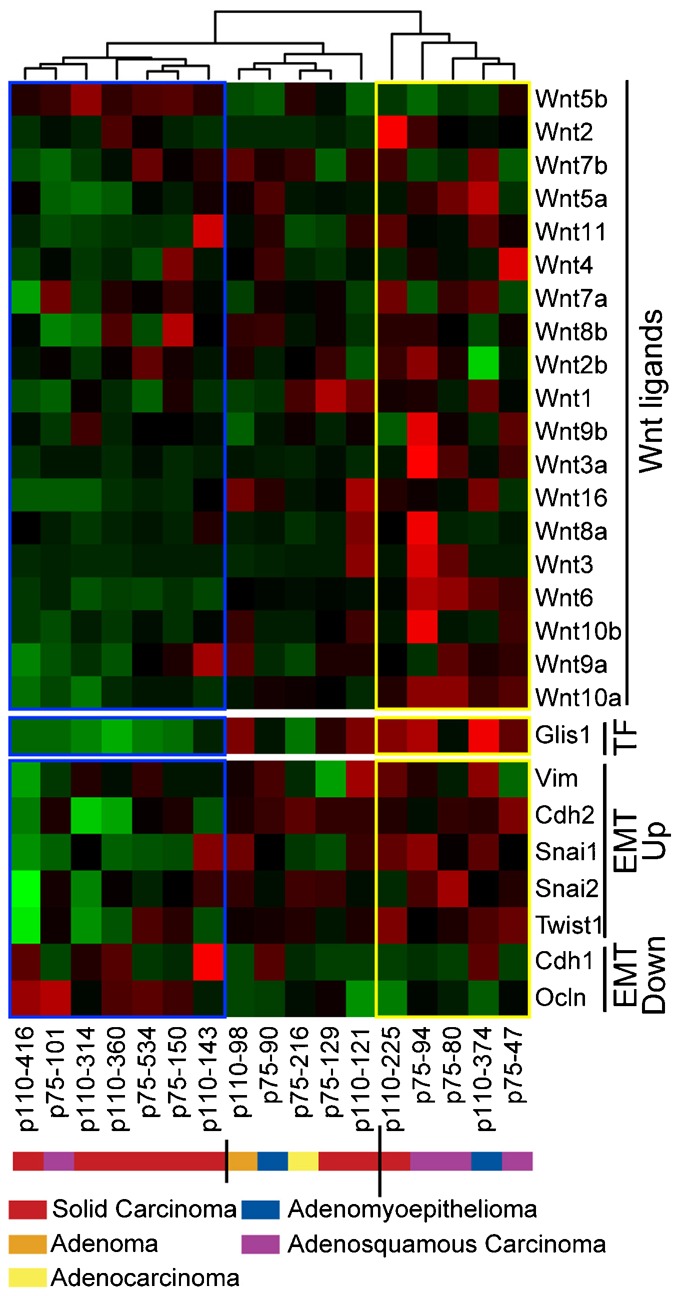
Activation of the Wnt/β-Catenin Pathway in MMTV-CUX1 Mammary Tumors Is Associated with High Expression of *Glis1*. Expression profiling was performed on microdissected tumor cells from 17 mammary tumors that developed in MMTV-p110 or p75 CUX1 transgenic mice, including 8 solid carcinomas (p110-416, p110-314, p110-360, p75-534, p75-150, p110-143, p75-129, p110-225), 4 adenosquamous carcinomas (p75-101, p75-94, p75-80, p75-47), 1 adenoma (p110-98), 2 adenomyoepitheliomas (p75-90, p110-374), and 1 adenocarcinoma (p75-216). The complete expression profiles are available on the Gene Expression Omnibus (GEO) repository under accession number GSE54804. The figure shows a heatmap of mammary tumors grouped according to an unsupervised hierarchical clustering of *Wnt* gene expression. Expression of *Glis1*, as well as *Vim*, *Cdh1*, *Cdh2*, *Snai1*, *Snai2*, *Ocln* and *Twist1* in each tumor is shown below. The cluster of “high-Wnt” tumors is boxed in yellow and the cluster of “low-Wnt” tumors is boxed in blue. Each column represents one tumor, with the transgene (p110 or p75 CUX1) and mouse ID number indicated below.

Since activation of the β-catenin pathway was observed in a fraction of mammary tumors from MMTV-CUX1 transgenic mice, we reasoned that it might require other transcription factors in addition to CUX1. As an approach to identify such transcription factors, we looked for factors whose expression was elevated in these mammary tumors. To ensure that changes in gene expression would not be masked by noise coming from stromal cells, mammary epithelial tumor cells were microdissected prior to performing expression profiling by microarray analysis. In total, 17 tumors of various histological types were analyzed and clustered using an unsupervised algorithm according to *Wnt* gene expression (Fig. 4). This procedure revealed three large clusters that displayed low, medium and high *Wnt* gene expression. Importantly, a distinct algorithm that linearly orders tumors over a gene signature (BreSAT) identified the same set of tumors as having the highest expression of *Wnt* genes (supplementary material Fig. S1). One gene coding for a transcription factor was clearly overexpressed in tumors with high Wnt gene expression: *Glis1*. In addition, we observed a correlation with genes whose expression is associated with epithelial-to-mesenchymal transition (EMT): *Vim*, *Cdh2*, *Snai1*, *Snai2* and *Twist1* were elevated, whereas *Cdh1* and *Ocln* were down-regulated (Fig. 4). In contrast, elevated *Wnt* gene expression did not correlate with that of β-Catenin mRNA, Wnt receptors or markers of stem and progenitor cells (supplementary material Fig. S2).

### Higher Expression of *WNT* Genes in Human Breast Cancers Correlates With *CUX1*, *GLIS1* and EMT Markers

**Fig. 5.**
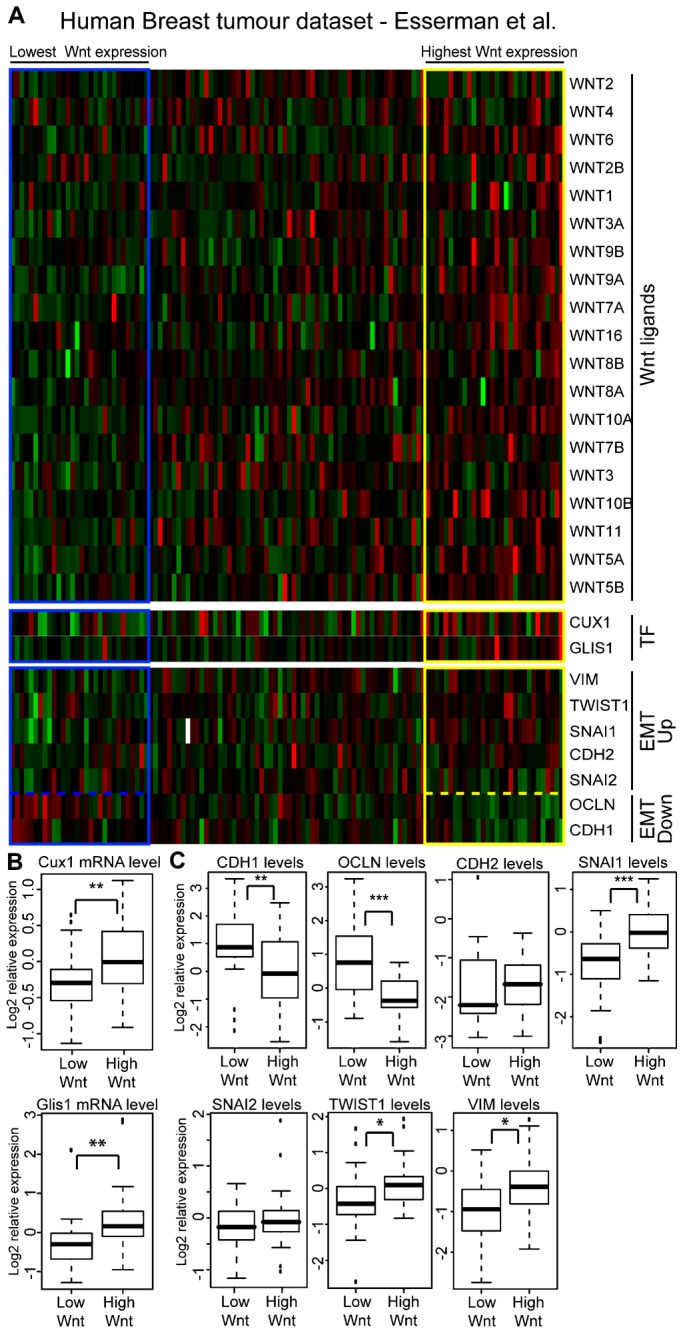
Correlation Between *WNT*, *CUX1* and *GLIS1* Gene Expression in a Human Breast Tumor Dataset. (A) Heatmap of a human breast tumor dataset sorted according to *WNT* genes expression using the BreSAT algorithm. Expression of *CUX1*, *GLIS1*, *VIM*, *TWIST1*, *SNAI1*, *CDH2*, *SNAI2*, *OCLN* and *CDH1* in each tumor is shown below. (B) *CUX1* and *GLIS1* expression in the top 25% and bottom 25% samples sorted according to *WNT* genes expression. * indicates p<0.05, ** <0.01, *** <0.001 on a Welch-corrected student's T test. (C) *CDH1*, *OCLN*, *CDH2*, *SNAI1*, *SNAI2*, *TWIST1* and *VIM* expression in the top 25% and bottom 25% samples sorted according to Wnt genes expression. * indicates p<0.05, ** <0.01, *** <0.001 on a Welch-corrected student's T test.

We verified whether high *WNT* gene expression correlates with that of *CUX1* and *GLIS1* in human breast cancers. As previously discussed, Affymetrix microarrays do not have a relevant probe for *CUX1* (Sansregret et al., 2011), but the Oncomine database still contained three breast cancer datasets with data on *CUX1* expression (Esserman et al., 2012; Glück et al., 2012; Cancer Genome Atlas Network, 2012). These datasets were sorted according to expression of *WNT* genes using the BreSAT algorithm. As mentioned above, this tool allows one to investigate a continuous trend across the data. The heatmap generated from one breast cancer dataset is presented in Fig. 5A as an example (Esserman et al., 2012). The top 25% of samples ranked according to *WNT* expression exhibited significantly higher expression of *CUX1* and *GLIS1* genes than the bottom 25% of samples (Fig. 5A,B). Overall, A correlation between *CUX1* and *WNT* levels was observed in the 3 out of 3 breast cancer datasets that had a relevant *CUX1* probe (Esserman, Gluck, TCGA), and between *Glis1* and *WNT* in 7 out of 8 datasets with Glis1 probes (supplementary material Table S1). In the Esserman dataset, we also observed a correlation with most, but not all, genes of the EMT signature: *VIM*, *SNAI1* and *TWIST1* were elevated, whereas *CDH1* and *OCLN* were decreased (Fig. 5A,C). A significant correlation between *WNT, CUX1, GLIS1* and EMT markers was also observed in the Gluck and the TCGA datasets (supplementary material Fig. S3).

### Ectopic Expression of GLIS1 in an MMTV-CUX1 Tumor Cell Line Leads to Wnt Genes Activation

**Fig. 6.**
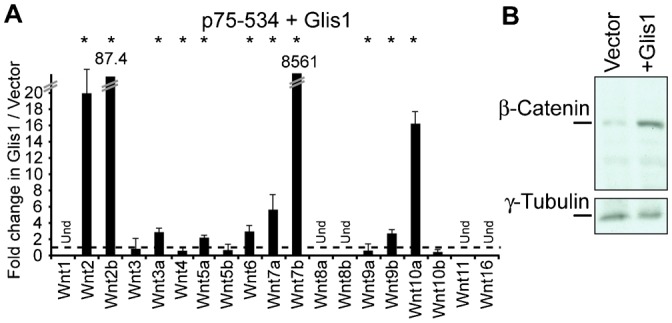
Glis1 expression in MMTV-CUX1 Tumor Cell line correlates to activation of the Wnt/β-Catenin pathway. (A) Cells of the “non-Wnt” p75-534 tumor line were infected with a lentivirus expressing GLIS1 or nothing (vector). Two days later, *Wnt* mRNA levels were measured by RT-qPCR. Und: Undetected. Results of triplicate experiments are shown. * P Value<0.05 on a Welch corrected T test. (B) β-Catenin expression was analyzed by immunoblotting in the same cells as in A.

Expression profiling analysis in human and mouse mammary tumors suggested that high *Wnt* gene expression requires both CUX1 and GLIS1. We verified this hypothesis using the “non-Wnt” cell line, p75-534, which displays low expression levels of *Wnt* and *Glis1* mRNA. To test whether higher GLIS1 expression was able to convert p75-534 cells to a “Wnt” phenotype, we ectopically expressed GLIS1 using a lentiviral vector and then measured the expression of *Wnt* mRNAs and β-catenin protein. We observed a 10-fold increase in the expression of *Wnt2*, *Wnt2b*, *Wnt7b* and *Wnt10a* and a 2-fold increase in *Wnt3a*, *Wnt5a*, *Wnt6*, *Wnt7a*, and *Wnt9b* (Fig. 6A). In agreement with these finding, the steady-state levels of β-catenin protein was significantly increased (Fig. 6B). We conclude that higher expression of GLIS1 in mammary tumor cells from MMTV-CUX1 transgenic mice leads to the transcriptional activation of several *Wnt* genes.

### GLIS1 and p110 CUX1 Cooperate to Activate the Wnt/β-Catenin Pathway and to Stimulate Cell Motility and Invasiveness

**Fig. 7.**
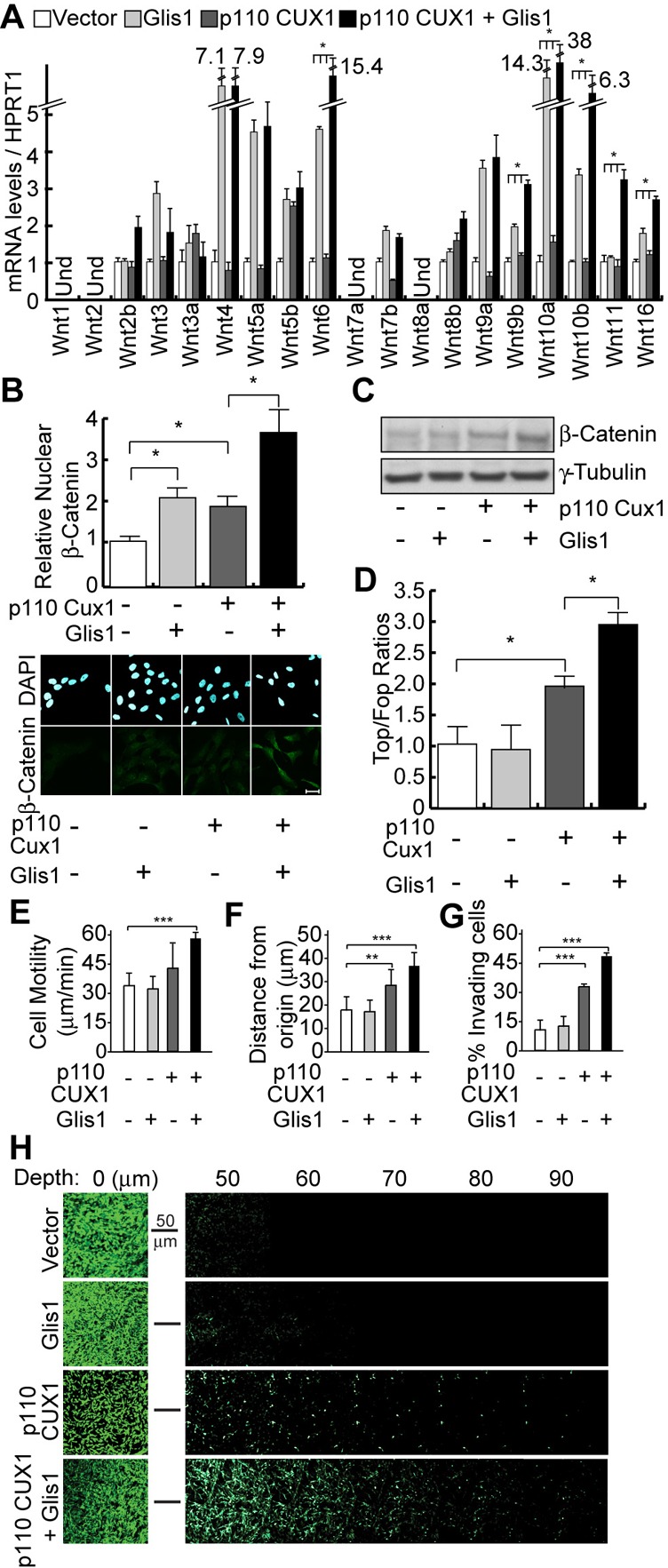
GLIS1 and p110 CUX1 Cooperate To Activate the Wnt/β-Catenin Pathway and to Stimulate Cell Motility and Invasiveness in Nontransformed Mammary Epithelial Cells. We established populations of MCF10A cells stably carrying lentiviral vectors that express nothing (vector), GLIS1, p110 CUX1, or both GLIS1 and p110 CUX1. (A) *Wnt* gene mRNA levels were measured by RT-qPCR. Und: Undetected. * P Value<0.05 on a Welch corrected T test when comparing the p110+Glis1 samples vs. each of the other 3. (B) β-Catenin protein levels were measured by immunofluorescence using antibodies specific for the active, non-phosphorylated form of the protein. Histogram shows the mean nuclear signal for β-Catenin for at least 50 cells per condition. * P Value<0.05 on a Welch corrected T test. A representative image of each condition is shown below. Scale bar: 20µm. (C) β-Catenin protein levels were measured by immunoblotting using antibodies specific for the active, non-phosphorylated form of the protein. γ-Tubulin is shown as a loading control. (D) MCF10A cells were transiently transfected with either the TOP of the FOP luciferase reporter, a vector expressing p110 CUX1 or an empty vector, and a vector expressing GLIS1 or the corresponding empty vector. Luciferase activity was measured in 3 independent transfections. * P Value<0.05 on a Welch corrected T test. (E) MCF10A cells ectopically expressing GLIS1, p110 CUX1 or both were imaged by time-lapse video microscopy and their speed of migration was quantified. The average of 3 independent experiments is shown with error bars representing standard error. ** P Value<0.01, *** <0.001 on 3 combined Welch corrected T tests using the Fisher method. (F) Time-lapse video microscopy was carried out as in E and distance migrated from the point of origin was quantified. (G) MCF10A cells overexpressing GLIS1 and p110 CUX1 alone or both were subjected to inverted Boyden chamber invasion assays. The percentage of cells able to migrate more than 20 µm into the matrix is shown for each line. The average of 3 independent experiments is shown. (H) Confocal Z-Stacks acquired 10 µm apart are aligned to demonstrate invasion of MCF10A-derivative cells into the matrix. Representative Z-stack for each condition is displayed. The leftmost image represents cells at the surface of the matrix (Depth = 0).

To verify whether GLIS1 and p110 CUX1 cooperate to activate the Wnt/β-catenin pathway, lentiviral expression vectors for either or both of these factors were introduced into MCF10A untransformed mammary epithelial cells derived from breast reduction surgery (Rak et al., 1991). Measurements of *WNT* gene expression revealed that each factor on its own is able to stimulate the expression of several *WNT* genes, however, for most genes highest expression was achieved when both GLIS1 and p110 CUX1 were co-expressed (Fig. 7A). In agreement with these results, highest levels of the non-phosphorylated nuclear β-catenin isoform (Fig. 7B,C) and highest TOP/FOP reporter activation (Fig. 7D) were observed in MCF10A cells stably expressing both GLIS1 and p110 CUX1.

To investigate whether the observed increase in *WNT* genes correlates with an EMT-like, migratory and invasive phenotype, we measured cell migration and invasion using live-cell imaging and a quantitative matrix invasion assays. Measurements of cell migration indicated that overexpression of GLIS1 alone was insufficient to enhance cell motility, that overexpression of p110 CUX1 promoted a moderate increase in cell migration, whereas overexpression of both GLIS1 and p110 CUX1 led to significant increases in both speed and distance of migration (Fig. 7E,F; supplementary material Fig. S4). To determine whether the combination of CUX1 and GLIS1 could promote cell invasion through a 3D matrix, we performed inverted invasion assays where cells are plated on the bottom of matrigel/collagen coated boyden chamber and are allowed to invade towards a chemo-attractant, serum in this case. As expected based on the live cell imaging results, we saw an increase in the invasive capacity of MCF10A cells co-expressing CUX1 and GLIS1 compared to either one alone (Fig. 7G,H).

**Fig. 8.**
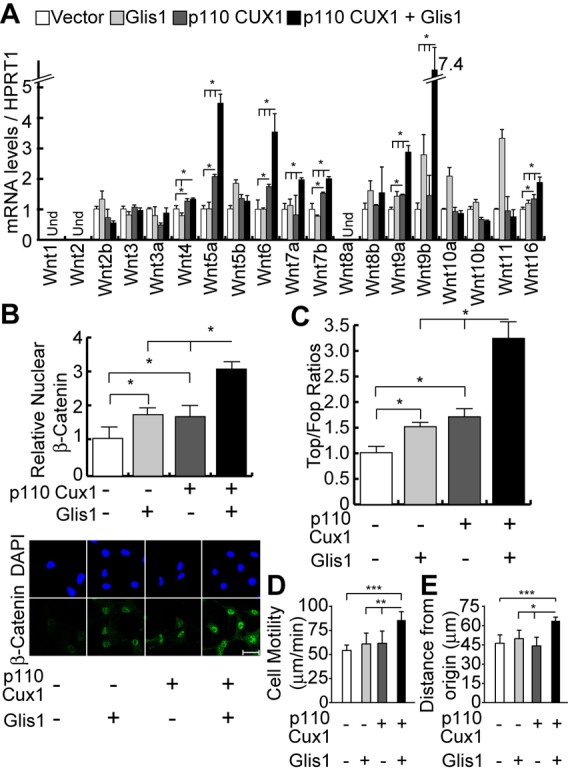
GLIS1 and p110 CUX1 Cooperate To Activate the Wnt/β-Catenin Pathway and to Stimulate Cell Motility in Breast Cancer Cells. We established populations of Hs578t cells stably carrying lentiviral vectors that express nothing (vector), GLIS1, p110 CUX1, or both GLIS1 and p110 CUX1. (A) Wnt gene mRNA levels were measured by RT-qPCR. Und: Undetected. * P Value<0.05 on a Welch corrected T test. (B) β-Catenin protein levels were measured by indirect immunofluorescence using antibodies specific for the active, non-phosphorylated form of the protein. Histogram shows the mean nuclear signal for β-Catenin for at least 50 cells per condition. * P Value<0.05 on a Welch corrected T test. A representative image of each condition is shown below. Scale bar: 20µm. (C) Hs578t cells were transiently transfected with either the TOP of the FOP luciferase reporter, and suboptimal amounts (50 ng) of effector plasmids coding for nothing (−), p110 CUX1 or GLIS1. Luciferase activity was measured in 3 independent transfections. * P Value<0.05 on a Welch corrected T test. (D) Hs578t cells ectopically expressing GLIS1, p110 CUX1 or both were imaged by time-lapse video microscopy and their speed of migration was quantified. The average of 3 independent experiments is shown with error bars representing standard error. ** P Value<0.01, *** <0.001 on a Welch corrected T test. (E) Time-lapse video microscopy was carried out as in D and distance migrated from the point of origin was quantified.

To verify whether p110 CUX1 and GLIS1 cooperate to activate the Wnt/β-catenin pathway in human breast cancer cells as well, Hs578t cells were infected with lentiviral vectors encoding either one of these factors or the combination of both. Combined expression of p110 CUX1 and GLIS1 had a significantly greater effect than either gene alone on the expression of several Wnt genes (Fig. 8A) and levels of nuclear β-catenin (Fig. 8B). TCF/β-catenin transcriptional activity was also tested using the TOP/FOP reporter system in cells co-transfected with suboptimal amounts of either the p110 CUX1 or GLIS1 effector plasmid, or the combination of both. The highest level of TOP/FOP ratio was observed in cells transfected with both effectors (Fig. 8C). Finally, a higher cell motility was measured in cells expressing both p110 CUX1 and GLIS1 (Fig. 8D,E). We conclude that p110 CUX1 and GLIS1 can cooperate in the activation of Wnt genes in human breast cancer cells.

## DISCUSSION

Strikingly, while there is a single Wingless gene in Drosophila, there are 19 Wnt genes in mammals. We understand that duplication of *Wnt* genes during evolution led to the association of *Wnt* coding sequences with a large repertoire of regulatory sequences, a process that ultimately enabled the expression of distinct *Wnt* genes in specific cells at precise times in order to activate Wnt pathways. Superimposed on the complex transcriptional regulation of Wnt ligands is the combinatorial nature of Wnt receptors and the multiple control steps that can prevent or limit Wnt pathway activation. Delivery of receptors at the cell membrane or secretion of ligands can be inhibited. Moreover, secretion of non-membrane bound receptors can squelch the action of ligands that are present in the extracellular milieu. Altogether, the multiple ways by which Wnt pathways can be activated and inhibited enables precise regulation both in time and space. Yet, perturbations in one or the other control systems have been found to cause aberrant activation of the Wnt/β-catenin pathway in human cancers. The majority of cases were reported in colon cancers and involve inactivating mutations of the APC tumor suppressor or mutations that affect axin or β-catenin itself (Segditsas and Tomlinson, 2006). In addition, autocrine activation of the Wnt/β-catenin pathway was shown to occur in a sizeable fraction of cancers from multiple organs and tissues (see Introduction). While elevated Wnt gene expression and repression of secreted Frizzled genes have been reported, we know very little about the transcription factors that play a role in the regulation of these genes.

In this study, we presented evidence from transgenic mouse models, tissue culture systems and human breast cancer databases implicating the CUX1 and GLIS1 transcription factors in the autocrine activation of the Wnt/β-catenin pathway. A fraction of mammary tumors from MMTV-p75 and p110 CUX1 transgenic mice exhibit higher Wnt gene expression (Cadieux et al., 2009). Using inhibitors that prevent activation of the FRP-LRP5/6 receptor in a mouse mammary tumor-derived cell line, we demonstrated that elevated *Wnt* gene expression leads to autocrine activation of the Wnt/β-catenin pathway (Fig. 1). CUX1 knockdown in six human tumor cell lines that display autocrine activation of the Wnt/β-catenin pathway showed that CUX1 is required for maximal expression of *WNT* genes (Fig. 2). In turn, ectopic expression of p110 CUX1 in three human cell lines led to increased β-catenin expression (Fig. 3). Moreover, stimulation of TCF/β-catenin transcriptional activity in this context was dependent on FRP-LRP5/6 receptor activation. Together these results clearly establish a causal link between CUX1 transcriptional function, the expression of *Wnt* genes, the activation of FRP-LRP5/6 receptors and the stimulation of TCF/β-catenin transcriptional activity. Note that we cannot exclude that Wnt ligands produced by a given cell in a culture dish can bind and activate a FZD/LRP receptor present on a neighboring cell, a type of signaling that could be described as paracrine. However, the term paracrine stimulation is reserved for cells of different types. Since cell lines maintained as monolayers in a culture dish are composed of one type of cells only, the term autocrine loop is more appropriate.

We note that distinct sets of Wnt genes were regulated in response to CUX1 knockdown or overexpression in different cell lines and tumors (Figs 2 and 4). For example, CUX1 did not stimulate the expression of WNT5A in MCF10A cells, although this gene was upregulated in two MMTV-CUX1 mammary tumors and was previously reported to be activated by CUX1 in the PANC1 and HT1080 cell lines (Figs 4 and 6) (Ripka et al., 2007). Clearly, CUX1 is neither sufficient nor required in all cell lines or tumors for the expression of all *Wnt* genes. A few points are worth considering. First, ChIP-chip assays in 8 different cell lines indicated that CUX1 binds to the promoter of only 6 Wnt genes: Wnt 1, 2, 4, 6, 8b and 10a (Cadieux et al., 2009; Harada et al., 2008). Although we cannot exclude that CUX1 may regulate other Wnt genes from a distant binding site, it is likely that CUX1 directly regulates only a subset of *Wnt* genes. The effect of CUX1 on other *Wnt* genes must be mediated indirectly through other transcription factors or co-regulators. We envision that precise regulation of each *Wnt* gene must rely on distinct combinations of transcription factors and co-regulators such that the ultimate effect of CUX1 on a particular *Wnt* gene will vary depending of the status of these factors in each cell line.

The fact that one third or less of mammary tumors from MMTV-CUX1 transgenic mice exhibited elevated *Wnt* gene expression indicated that CUX1 alone is not sufficient to activate this pathway and provided an opportunity to identify other transcription factors that cooperate with CUX1 in this process. Expression profiling of microdissected mammary epithelial tumor cells revealed that *Glis1* is overexpressed in tumors that display elevated *Wnt* expression (Fig. 4). Meta-analysis of human breast cancer datasets showed that elevated *WNT* gene expression also correlated with high levels of *CUX1* and *GLIS1* (Fig. 5; supplementary material Fig. S3; Table S1). High expression of GLIS1 in cancers is particularly striking considering that *Glis1* expression in mouse was found to be elevated in unfertilized eggs and one-cell embryos but weak in adult tissues (Maekawa et al., 2011). Co-expression experiments in the MCF10A non-transformed mammary epithelial cell line confirmed that CUX1 and Glis1 can cooperate to activate several Wnt genes and stimulate TCF/β-catenin transcriptional activity (Fig. 7).

GLIS1 has been shown to cooperate with Oct3/4, Sox2 and Klf4 (OSK) in the activation of several *Wnt* genes and in the reprogramming of mouse fibroblasts (Maekawa et al., 2011). Expression profiling analysis indicated that GLIS1 cooperates with OSK to increase the expression of several Wnt ligands (Wnt3, Wnt6, Wnt8a and Wnt10a) in p53^−/−^ MEFs. We also observed that that GLIS1 increased the expression of many Wnt genes in MCF10A cells: Wnt3, Wnt4, Wnt5b, Wnt6, Wnt9a, Wnt10a and Wnt10b (Fig. 7A). We did not, however, observe the upregulation of stem or progenitor cell markers in mammary tumors that otherwise displayed Glis1, CUX1 and Wnt expression (supplementary material Fig. S2). Hence, we conclude that Glis1 is not sufficient but requires OSK factors to activate stem cell markers. One functional class of genes whose regulation in mammary epithelial cells correlated with that of *Wnt*, *Cux1* and *Glis1* are those associated with epithelial-to-mesenchymal transition (EMT). Both in mouse mammary tumors and in human breast cancers, we observed elevation of Vim, Cdh2 (N-cadherin), Snai1 and Twist1 and downregulation of Cdh1 (E-cadherin) and Ocln (Figs 4, 5; supplementary material Fig. S3). These findings are in agreement with previous studies showing that *CUX1* knockdown inhibits cell motility (Michl and Downward, 2006; Michl et al., 2005) and that ectopic CUX1 expression can stimulate cell migration and invasion (Kedinger and Nepveu, 2010; Kedinger et al., 2009). Our results in MCF10A human mammary epithelial cells revealed that GLIS1 cooperates with CUX1 to simulate cell migration and invasion. Altogether, the findings that GLIS1 stimulates both the production of induced pluripotent stem cells by OSK factors and the activation of EMT markers by CUX1 indicate that GLIS1 can regulate distinct sets of transcriptional targets depending on cell context. Overall, results from these two independent studies reveal an important role of GLIS1 in reprogramming gene expression profiles.

Activation of Wnt signaling has been associated with an EMT gene expression signature in a number of studies, notably in basal-like breast cancers (DiMeo et al., 2009). Whether activation of Wnt signaling is sufficient by itself to promote cell motility is not clear. We can envisage a scenario where both Wnt signaling and cell motility would be stimulated by a common set of transcription factors. Indeed, the present study strongly suggests that GLIS1 and CUX1 play an important role in the promotion of both the Wnt signaling and the EMT phenotype in many cancer cells.

## Supplementary Material

Supplementary Material
