# Supplementary material for: Autocrine Activation of the Wnt/β-Catenin Pathway by CUX1 and GLIS1 in Breast Cancers
Source: Biol Open. 2014 Sep 12;3(10):937–46. doi: 10.1242/bio.20148193 (PMC4197442; doi:10.1242/bio.20148193)
Supplement: Supplementary Material [file supp_3_10_937__index.html]

Autocrine Activation of the Wnt/β-Catenin Pathway by CUX1 and GLIS1 in Breast Cancers — Supplementary Material 

# Autocrine Activation of the Wnt/β-Catenin Pathway by CUX1 and GLIS1 in Breast Cancers

## bio.20148193 Supplementary Material

**Files in this Data Supplement:**

- Supplementary Material - Charles Vadnais et al. doi: 10.1242/bio.20148193
