# Supplementary material for: Autocrine Activation of the Wnt/β-Catenin Pathway by CUX1 and GLIS1 in Breast Cancers
Source: Biol Open. 2014 Sep 12;3(10):937–46. doi: 10.1242/bio.20148193 (PMC4197442; doi:10.1242/bio.20148193)
Supplement: Supplementary Material [file supp_bio.20148193_bio.20148193-s1.pdf]

Biology Open (2014) 000, 1–10 doi:10.1242/bio.20148193

Supplementary Material
Charles Vadnais et al. doi: 10.1242/bio.20148193

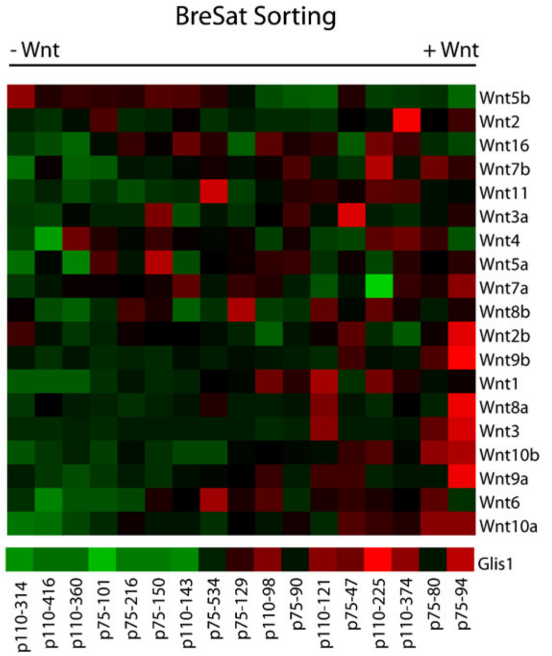

Fig. S1. BreSAT sorting of MMTV-CUX1 Mammary Tumors. Heatmap representation of mammary tumors ranked according to Wnt expression using the BreSAT algorithm.

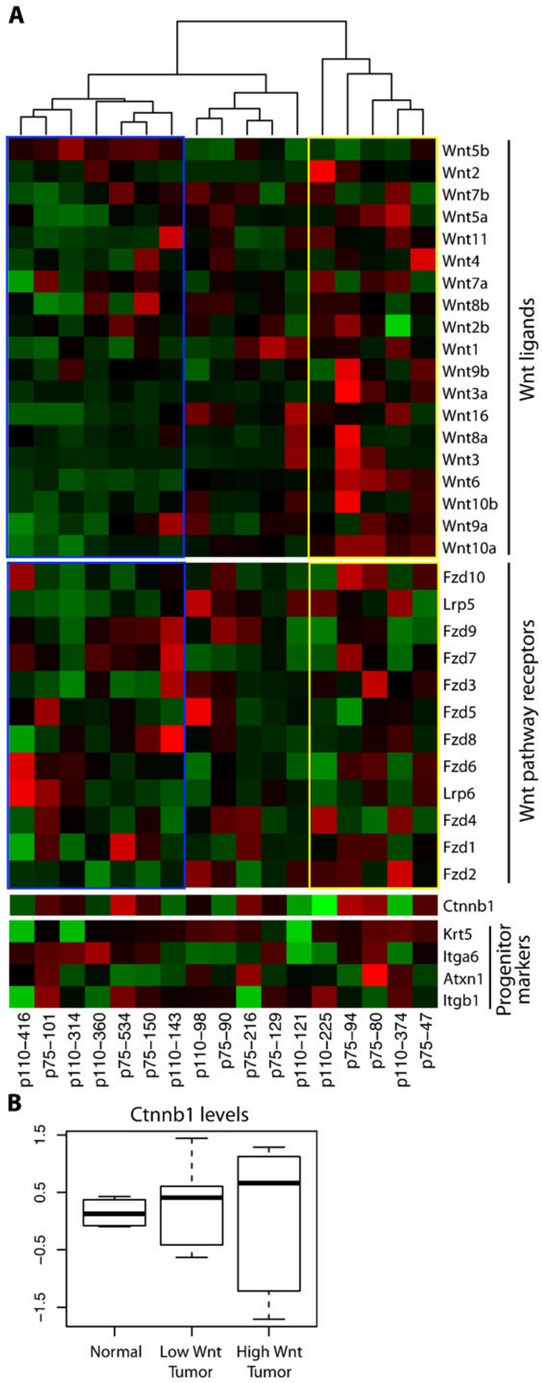

Fig. S2. Activation of the Wnt/β-Catenin Pathway in MMTV-CUX1 Mammary Tumors is independent of Frzd receptor and β-Catenin transcript levels. (A) Heatmap of mammary tumors clustered according to Wnt gene expression, with corresponding expression of Frzd receptor genes, β-Catenin and markers of progenitor cells. (B) Boxplot representation of β-Catenin expression in normal mammary epithelial cells, ''non-Wnt'' tumors and ''Wnt'' tumors.

Biology Open

Biology Open (2014) 000, 1–10 doi:10.1242/bio.20148193

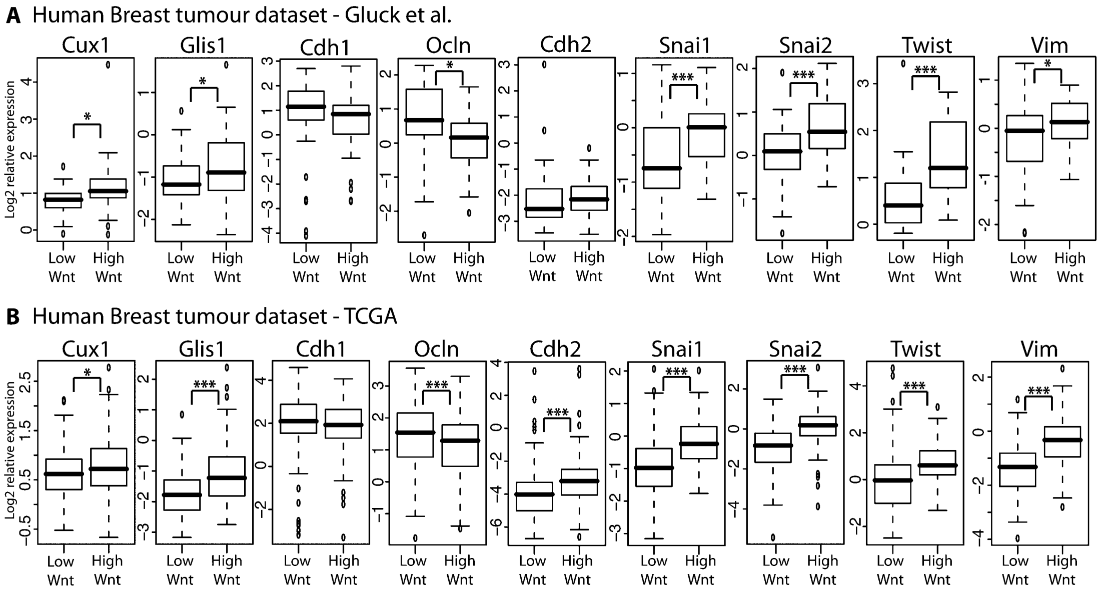

Fig. S3. Expression of CUX1, Glis1 and EMT Markers in Human Breast Cancer Datasets. (A) Expression of indicated genes in the top 25% (High Wnt) and bottom 25% (Low Wnt) samples sorted according to Wnt genes expression from the Glück et al. human breast cancer dataset from Oncomine (Glück et al., 2012). * indicates $p<0.05$, **<0.01, *** <0.001 on a Welch-corrected student's T test. (B) Analysis as in A for the TCGA Dataset (Cancer Genome Atlas Network, 2012).

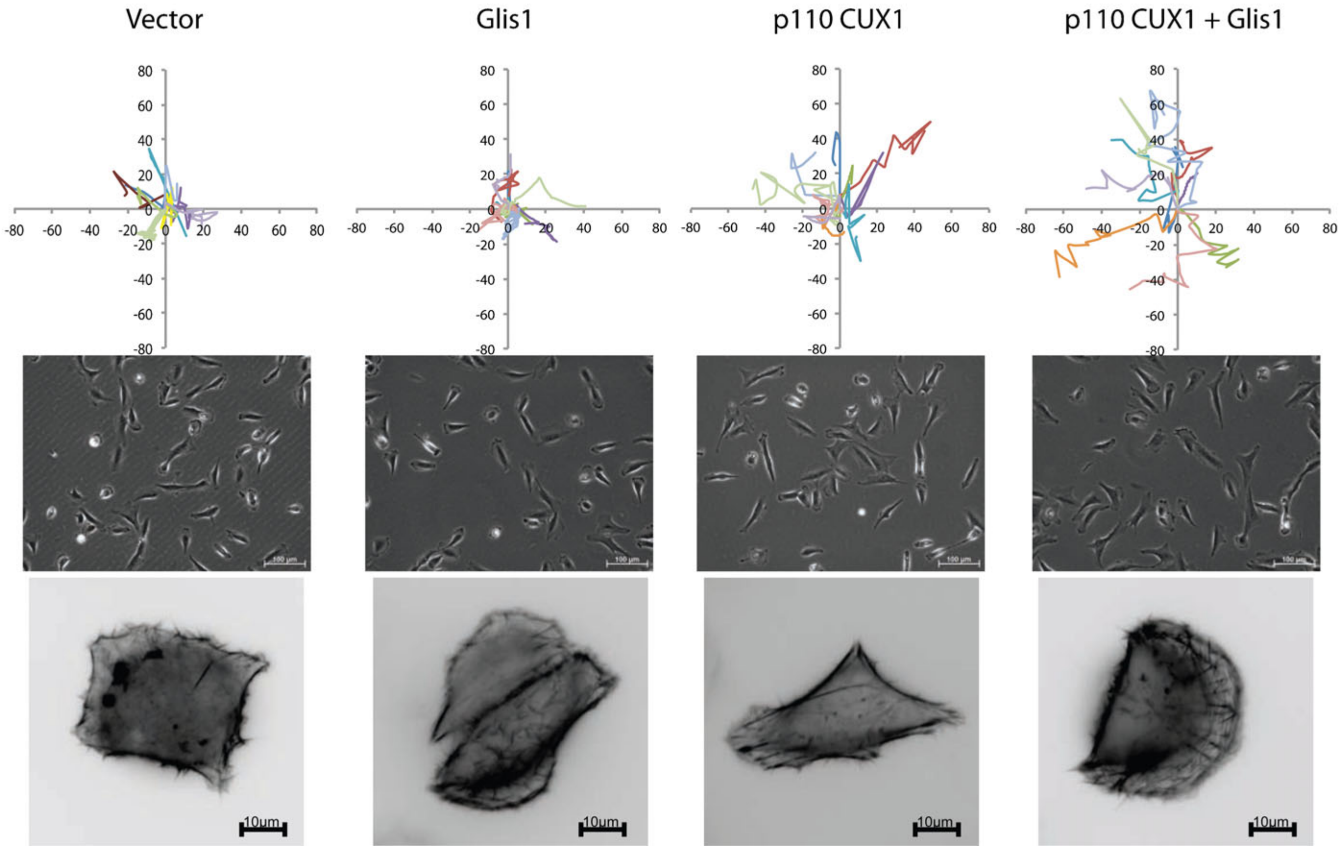

Fig. S4. Tracking Plot of Cell Migration and Sample Images. MCF10A cells over expressing Glis1, p110 CUX1 or both were imaged by time-lapse video microscopy (Fig. 7). Ten representative cells from each cell line were randomly selected and their migratory track relative to the origin is depicted using the X and Y coordinates (µm). Representative phase contrast images of the cells are also shown for each cell line. MCF10A cells expressing vector, Glis or p110 CUX1 alone had an elongated morphology with very little to no ruffles and no defined leading edge. Whereas in the presence of Glis and p110 CUX1, cells form large lamellapodial ruffles at the leading edge. Scale bars: 100 µm (middle), 10 µm (bottom).

Biology Open (2014) 000, 1–10 doi:10.1242/bio.20148193

Table S1. Correlation between Wnt, CUX1 and GLI gene expression in human tumor datasets

Gene	Breast cancer datasets
CUX1	3 of 3 (100%)
Glis1	7 of 8 (88%)

The table shows the number of human breast cancer datasets retrieved from Oncomine in which the top 25% of samples ranked according to Wnt gene expression display significantly higher expression of *CUX1* or *GLIS1* than the bottom 25% of samples, as tested by a Welch corrected T Test with $P < 0.05$.

Biology Open
